# Cardiorespiratory fitness and health-related quality of life in survivors of childhood central nervous system tumours

**DOI:** 10.1007/s00520-023-07854-9

**Published:** 2023-06-15

**Authors:** Rachael Keating, Sarah Curry, Juliette Hussey

**Affiliations:** 1 School of Medicine, Trinity College Dublin, University of Dublin, Dublin, Ireland; 2 Physiotherapy Department, Children’s Health Ireland at Crumlin, Dublin, Ireland; 3 National Children’s Cancer Service, Children’s Health Ireland at Crumlin, Dublin, Ireland; 4 School of Medicine, University College Dublin, Dublin, Ireland

**Keywords:** Cardiorespiratory fitness, Quality of life, Central nervous system tumour, Paediatric, Survivorship

## Abstract

**Purpose:**

We assessed cardiorespiratory fitness and health-related quality of life (HRQoL) in survivors of childhood central nervous system (CNS) tumours.

**Methods:**

Participants were recruited from the National Children’s Cancer Service in Children’s Health Ireland at Crumlin. Inclusion criteria included diagnosis of a primary CNS tumour, aged between 6 and 17 years, between 3 months and 5 years post completion of oncology treatment, independently mobile, and deemed clinically appropriate to participate by treating oncologist. Cardiorespiratory fitness was assessed using the six-minute walk test. HRQoL was assessed with the PedsQL Generic Core Scales, Version 4.0.

**Results:**

Thirty-four participants (*n* = 16 male) were recruited, with a mean age of 12.21 ± 3.31 years and a mean time since completion of oncology treatment of 2.19 ± 1.29 years. Mean six-minute walk distance (6MWD) achieved was 489.56 ± 61.48 m, equating to the 8^th^ percentile overall. 6MWD was significantly reduced when compared to predicted population norms (*p* < 0.001). PedsQL parent proxy-report and child-report scores were significantly lower when compared to healthy paediatric norms (*p* < 0.001 – *p* = 0.011). A significant positive correlation was found between 6MWD and both parent proxy-report (r = 0.55, *p* < 0.001) and child-report (r = 0.48, *p* = 0.005) PedsQL total scores.

**Conclusion:**

Survivors of childhood CNS tumours present with impaired cardiorespiratory fitness and HRQoL. Higher levels of cardiorespiratory fitness are associated with higher levels of HRQoL.

**Implications for Cancer Survivors:**

Routine screening of cardiorespiratory fitness and HRQoL in survivors of childhood CNS tumours may be beneficial. Healthcare providers should encourage and provide education on the potential benefits of physical activity to improve overall quality of life.

**Supplementary Information:**

The online version contains supplementary material available at 10.1007/s00520-023-07854-9.

## Introduction

Tumours involving the central nervous system (CNS) are the second most common type of childhood cancer, accounting for approximately 22.6% of all childhood cancer cases [1]. Recent advances and improvements in surgical techniques, imaging and adjuvant therapies have led to improved survival rates for children with CNS tumours [2, 3]. These improvements in survival rates have resulted in more emphasis now being placed on exploring and reducing late-effects, cognitive and physical rehabilitation and enhancing the independence of long-term survivors of childhood CNS tumours.

Longer survival can come at a great cost, with the majority of all survivors of childhood cancer experiencing at least one treatment-related late effect [4–6]. In survivors of childhood CNS tumours, the majority of the late-effects experienced can be directly attributed to neurological damage caused by the original tumour, neurosurgical interventions, chemotherapy toxicities and the effects of radiation on the CNS [7]. Known late-effects amongst survivors of childhood CNS tumours include endocrinopathies, obesity, cerebrovascular complications, neurologic conditions, neurocognitive dysfunction and cardiovascular and pulmonary dysfunction, all of which can negatively impact on overall quality of life [7–12]. Survivors of childhood CNS tumours along with survivors of bone sarcomas are known to have the highest prevalence of functional limitations amongst all survivors of childhood cancer [13].

CNS tumour pathologies are diverse and can range from low to high grade lesions and include benign and malignant tumours [9]. Studies exploring the physical and psychosocial late-effects of childhood CNS tumours tend to focus on children with a diagnosis of high-grade tumours; however, there is evidence to suggest that these late-effects are not restricted to childhood survivors of high-grade tumours and can also be experienced by children treated for low-grade tumours [14, 15]. Long-term survivors of low-grade tumours such as circumscribed astrocytic glioma can experience considerable morbidity which can have the capacity to significantly alter their overall quality of life in survivorship [9].

A recent meta-analysis found that survivors of childhood cancer engage in significantly less physical activity and have significantly lower levels of fitness than a non-cancer control group [16]. The majority of studies examining cardiorespiratory fitness in childhood cancer survivors focus primarily on survivors of leukaemia and include low numbers of survivors of CNS tumours or often analyse survivors of CNS tumours as part of a mixed cancer diagnosis group [17–20]. This makes it difficult to determine the levels of cardiorespiratory fitness specifically amongst survivors of childhood CNS tumours.

Survivors of childhood CNS tumours have been found to have lower levels of cardiorespiratory fitness than their same aged peers and also than other children with chronic health conditions such as cystic fibrosis [21]. Hoffman et al. [17] similarly found that survivors of childhood CNS tumours present with significantly lower levels of cardiorespiratory fitness than a sibling control group. Lower levels of physical activity have also been found to be associated with reduced cardiorespiratory fitness amongst survivors of childhood CNS tumours [21, 22]. Whilst each of these three studies used validated objective outcome measures, a particular weakness of these studies was the small number of survivors of childhood CNS tumours included, with a total across all three studies of 52 survivors [17, 21, 22]. These small subject numbers make the results of these studies difficult to generalise to the overall population of survivors of childhood CNS tumours and therefore more data is needed to accurately describe cardiorespiratory fitness in this cohort.

With the more recent intensified research focus on quality of survivorship outcomes for childhood cancer survivors, more and more evidence is available to show that this cohort experience significantly lower levels of mental, emotional, social and physical functioning than their healthy peers [23–28]. Risk factors linked to adverse health-related quality of life (HRQoL) and mental health outcomes have been identified and include female gender, lower educational level, lower socioeconomic status and being diagnosed with cancer during adolescence [23, 24].

Children diagnosed with CNS tumours are especially vulnerable in experiencing reduced psychosocial functioning and quality of life given that they are diagnosed and undergo intensive treatment during a period of rapid physical, psychological and social growth and change. Neurocognitive impairments are common amongst survivors of childhood CNS tumours, with many papers also reporting lower rates of academic achievement and increased rates of special education service use amongst this cohort [29–32]. In adulthood, survivors of childhood CNS tumours experience higher rates of unemployment, social isolation and are at increased risk for being unable to live independently [33].

Health-related quality of life is a multidimensional concept and refers to the subjective view of the individual person about their own life situation. It is considered to include the following domains: physical, psychological and social health [34]. HRQoL has recently emerged as a fundamental health outcome in paediatric CNS tumour clinical trials [35–37]. There is a growing body of evidence that suggests survivors of childhood CNS tumours present with lower levels of HRQoL when compared to other childhood cancer survivors or their healthy peers [38]. Looking specifically at the different domains within HRQoL, survivors of childhood CNS tumours have been found to report significantly lower levels of physical functioning than survivors of childhood leukaemias, lymphomas and sarcomas [39]. Data available on HRQoL amongst survivors of childhood CNS tumours can be conflicting, with some studies reporting levels of HRQoL within normal ranges [40, 41], although favourable results tend to often be related to a specific CNS tumour diagnosis such as a circumscribed pilocytic astrocytoma [42].

This cross-sectional study aimed to measure cardiorespiratory fitness and HRQoL in survivors of childhood CNS tumours and to determine whether associations exist between cardiorespiratory fitness, HRQoL and clinical characteristics.

## Methods

### Participants

Participants were recruited from September 2021 to March 2022 through the neuro-oncology clinic in the National Children’s Cancer Service based at Children’s Health Ireland at Crumlin, Ireland. Inclusion criteria included children who had completed treatment for a childhood CNS tumour at least 3 months prior to study recruitment, aged between 6 and 17 years, independently mobile with or without an aid, engaged in ongoing long-term follow up care through the Neuro-Oncology clinic and deemed clinically well enough to participate by their primary treating oncologist. Children were excluded if they had completed treatment for a childhood CNS tumour greater than 5 years before study recruitment and/or had any significant comorbidities (e.g. significant physical disability, significant cognitive impairment) that would limit their ability to complete objective testing.

### Measures

#### Cardiorespiratory fitness

The six-minute walk test (6MWT) was used to assess cardiorespiratory fitness levels amongst participants. The 6MWT has been found to be both valid and reliable in paediatric populations and has been previously used in the paediatric cancer setting [19, 43, 44]. The 6MWT was conducted along a 20-m flat track. The assessment was conducted by a chartered physiotherapist who was a member of the research study team and administered as per the American Thoracic Society guidelines [45]. Standardised instructions were given throughout the process for each subject to ensure uniformity in testing procedure. The following measurements were collected: total distance walked, pre-test and post-test measures of heart rate, oxygen saturation and rate of perceived exertion using BORG’s 1–10 scale [46]. The equations developed by Ulrich et al. [47] were used to calculate age and sex-matched predicted 6-min walk distances (6MWD) for each participant (See Online Resource Table 1). The observed total distance covered was then compared to age and sex-matched predicted distance values [47]. The age-dependent percentiles for 6MWD in healthy children and adolescents were used to calculate individual percentile ranks for our study population [47].

#### Health-related quality of life

Health-related quality of life (HRQoL) was measured using the Paediatric Quality of Life Inventory (PedsQL) Generic Core Scales, version 4.0. The PedsQL is valid and reliable for use with paediatric cancer populations [48, 49]. The questionnaire consists of 23 different questions, which assess HRQoL across the domains of physical, emotional, social and school functioning. Parents completed the parent-report form and study participants completed the child-report forms. Younger children were provided with assistance in completing their questionnaire, when needed, by the principal researcher. Questionnaire scores were converted to scale scores between 0 and 100, with higher scores representing higher levels of HRQoL. Along with the total scale score, a physical scale score and a psychosocial scale score were also obtained. The psychosocial scale score is obtained from the combined total of the emotional, social and school functioning domains.

### Procedure

This was a cross-sectional, observational study. Initial screening was completed by a member of the research team and the primary treating oncologist. Eligible participants were contacted by post and provided with the relevant participant information leaflets prior to their neuro-oncology clinic visit, with final written informed consent and assent provided then by parents/guardians and participants in-person at their clinic appointment. Ethical approval for this study was granted by the research ethics committees in Children’s Health Ireland (GEN/935/21) and Trinity College Dublin (Ref 210,508).

Testing was scheduled to coincide with each participant’s neuro-oncology clinic appointment. Participants and one parent/guardian per participant first completed the HRQoL questionnaires, followed by the 6MWT. The testing procedure typically took thirty minutes to complete.

Demographic variables collected included gender, participant’s age, age at diagnosis and time since completion of treatment. Anthropometric measures including height and weight were measured and recorded. Cancer-related variables collected included diagnosis, presence of hydrocephalus at diagnosis, requirement for surgical management of hydrocephalus and oncology treatments received (surgery, chemotherapy, radiation therapy).

### Statistical analysis

Descriptive statistics were used to summarise patient characteristics. Mean and standard deviation were used to describe continuous variables and frequency and percentage were used to describe categorical variables. The Shapiro–Wilk test was used to examine the distribution of continuous variables.

Paired *t*-tests and one sample *t*-tests were used to compare the mean differences between 6MWD and PedsQL scale scores with predicted values and healthy paediatric population norms [47, 48]. Additionally, Cohen’s D was calculated to measure the effect size of the difference between the observed 6MWD achieved by our study population and the age and sex-matched predicted 6MWD for our population. Effect sizes were similarly calculated to measure the difference between the PedsQL scores of our study population and healthy population norms. Effect sizes were interpreted according to Cohen [50], with effect sizes between 0.2 and 0.5 considered as small, effect sizes between 0.5 and 0.8 as moderate and effect sizes ≥ 0.8 considered large.

*T*-tests and one-way analyses of variance were used as appropriate, to examine the associations between cardiorespiratory fitness and HRQoL with demographic and oncology treatment categorical variables. The associations between cardiorespiratory fitness and HRQoL with demographic continuous variables were examined using Pearson correlations. Pearson correlations were used to examine the association between cardiorespiratory fitness and HRQoL.

IBM Statistics SPSS Version 27 was used to conduct all statistical analyses. Significance level was set at a two-sided *P* value of 0.05 for all analyses. Results are shown as mean ± standard deviation unless otherwise stated.

## Results

Of the 38 participants who were deemed eligible following initial screening and invited to participate in the study, 34 participants consented to participate, giving a recruitment rate of 89.47%. Amongst the 34 survivors of childhood CNS tumours recruited, 16 were male (47.06%) with a mean age of 12.21 ± 3.31 years. The mean age at diagnosis was 9.09 ± 4.27 years and mean time since completion of oncology treatment was 2.19 ± 1.29 years.

CNS tumours were classified into groups in line with the 2021 WHO Classification of Tumours of the Central Nervous System. [51] The two most prevalent types of childhood CNS tumour were circumscribed astrocytic gliomas (32.35%) and medulloblastomas (26.47%). The detailed characteristics of the participants are summarised in Table 1.

**Table 1:** Participant characteristics (*n* = 34)

	Mean (SD) or frequency (%)
Gender	
Male	16 (47.406%)
Female	18 (52.94%)
Age at diagnosis (years)	9.09 (4.27)
Age at study participation (years)	12.21 (3.31)
BMI Z-score	0.27 (1.25)
Time since treatment completion (years)	2.19 (1.29)
Tumour type	
Circumscribed astrocytic glioma	11 (32.35%)
Medulloblastoma	9 (26.47%)
Glioma, glioneuronal & neuronal tumours	6 (17.65%)
Ependymal tumours	4 (11.76%)
Germ cell tumour	2 (5.88%)
Glioblastoma	1 (2.94%)
Pineal tumours	1 (2.94%)
Hydrocephalus at diagnosis	
Present	19 (55.90%)
Not present	15 (44.10%)
Permanent CSF diversion therapy	
ETV	1 (2.90%)
VP shunt	12 (35.30%)
Nil	21 (61.80%)
Treatment	
Surgery	34 (100%)
• Biopsy only	4 (11.76%)
• Sub-total resection	3 (8.82%)
• Near-total resection	8 (23.53%)
• Gross-total resection	19 (55.88%)
Chemotherapy	19 (55.88%)
Radiation therapy	15 (44.12%)
• Focal	3 (8.82%)
• Craniospinal + local boost	12 (35.29%)
Treatment combinations	
Surgery only	12 (35.30%)
Surgery + chemotherapy	6 (17.60%)
Surgery + radiation therapy	4 (11.80%)
Surgery + chemotherapy + radiation therapy	12 (35.30%)

*CSF*, cerebrospinal fluid; *BMI*, body mass index; *ETV*, endoscopic third ventriculostomy; *SD*, standard deviation; *VP shunt*, ventriculoperitoneal shunt

### Cardiorespiratory fitness

Thirty-four participants completed the 6MWT. No participants required the use of supplemental oxygen during or after the 6MWT, one participant used a mobility aid to complete the assessment and there were no adverse events during the testing.

Participants achieved a mean distance of 489.56 ± 61.48 m (370–680 m) on the 6MWT, equating to a rank on the 8^th^ ± 13.49^th^ percentile overall [47]. There was a significant difference between 6MWD achieved and the sex and age-adjusted predicted 6MWD for our population (*p* < 0.001, CI [-152.51, -108.96]), with our study population achieving a significantly lower overall distance on the 6MWT (see Table 2). A significantly large negative effect size was found (d = -2.10) when measuring the difference in the observed 6MWD of our study population and the sex and age-adjusted predicted 6MWD for our population.

**Table 2:** Six-minute walk distances in metres

	Observed 6MWD Mean (SD)	Predicted 6MWD Mean (SD)	*p*-value
Total population (*n* = 34)	489.56 (61.48)	620.29 (42.17)	< 0.001
CNS tumour groups			0.013
Group 1 (*n* = 17)	499.12 (51.97)	623.29 (36.66)	
Group 2 (*n* = 9)	442.22 (47.64)	623.11 (47.86)	
Group 3 (*n* = 8)	522.50 (68.61)	610.75 (50.59)	

*6MWD*, 6 minute walk distance; *SD*, standard deviation; *CI*, 95% confidence interval

CNS tumour groups:

Group 1 Gliomas, glioneuronal and neuronal tumours

Group 2 Medulloblastoma

Group 3 Other central nervous system tumours

CNS tumours were divided into three different groups to allow for comparisons of cardiorespiratory fitness outcomes across different CNS tumour types. The first group included gliomas, glioneuronal and neuronal tumours (*n* = 17), the second group included medulloblastoma (*n* = 9) and all other CNS tumours (*n* = 8) were placed into the third group. A significant difference was found in cardiorespiratory fitness across the different tumour groups (*p* = 0.013), with survivors of medulloblastoma presenting with lower levels of cardiorespiratory fitness than survivors of gliomas, glioneuronal and neuronal tumours (*p* = 0.046) and other CNS tumours (*p* = 0.014) (see Table 2).

No significant differences were found to exist between age (*p* = 0.075, 95% CI [-0.03, 0.59]), time since treatment completion (*p* = 0.895, 95% CI [-0.32, 0.36]), and BMI Z-score (*p* = 0.867, 95% CI [-0.31, 0.36]) with cardiorespiratory fitness levels. Cardiorespiratory fitness also did not differ between participants who underwent different medical, surgical and oncology treatments. See Table 3 for a detailed breakdown of results comparing cardiorespiratory fitness outcomes with participant clinical characteristics.

**Table 3:** Comparisons of six-minute walk distance and PedsQL scores with participant clinical characteristics

	6MWD Mean difference (CI)	PedsQL child Mean difference (CI)	PedsQL parent Mean difference (CI)
Gender	-0.35 (-44.04, 43.35)	2.18 (-7.42, 11.77)	-4.15 (-14.69, 6.38)
Presence of hydrocephalus	-0.19 (-44.12, 43.73)	2.02 (-7.66, 11.69)	1.50 (-9.19, 12.18)
Permanent CSF diversion therapy	0.03^*a*^	0.11 ^*a*^	0.74 ^*a*^
CNS tumour type	5.00*^a^	1.11^*a*^	3.55*^a^
Chemotherapy	-15.11 (-58.69, 28.48)	-0.70 (-10.40, 9.00)	-6.21 (-16.67, 4.25)
Radiation therapy	-23.07 (-66.20, 20.06)	-6.85 (-16.15, 2.44)	-7.85 (-18.16, 2.47)
Oncology treatment combinations	0.663^*a*^	1.82^*a*^	3.20*^a^

*6MWD*, 6-minute walk distance; *CI*, confidence interval; *CSF*, cerebrospinal fluid; *PedsQL*, Pediatric Quality of Life Inventory Generic Core scales

Results reported from independent *t*-tests unless otherwise indicated

^*^ Indicates significant result, *p* < 0.05

^*a*^ Result reported is from one-way analysis of variance

### Health-related quality of life

Thirty-four parents/guardians completed the parent proxy-report PedsQL questionnaires and 33 study participants completed the self-report PedsQL questionnaires. The PedsQL child and parent-reported total and subscale scores are presented in Table 4, with comparisons made to scores from a healthy normative paediatric population [48]. Mean PedsQL total scores were lower than the normative population scores for both self-reported and parent proxy-reported HRQoL.

**Table 4:** PedsQL generic core scale scores and comparison with normative population

PedsQL generic core scales	Study population	Normative population	*p*-value^*a*^	Effect size^*b*^
Self-report (n = 33)	Mean (SD)	Mean (SD)		
Total score	76.78 (13.29)	83.00 (14.79)	0.011*	-0.47
Psychosocial health	76.56 (14.15)	82.38 (15.51)	0.024*	-0.41
Physical health	77.18 (15.61)	84.41 (17.26)	0.012*	-0.46
Parent proxy-report (*n* = 34)				
Total score	67.21 (14.97)	87.61 (12.33)	< 0.001*	-1.36
Psychosocial health	67.50 (14.02)	86.58 (12.79)	< 0.001*	-1.36
Physical health	66.68 (19.46)	89.32 (16.35)	< 0.001*	-1.16

Key: ^*a*^* P*-values calculated for between group comparisons of PedsQL scores between study population and normative population data using independent *t*-tests

^*b*^ Effect size: reported as Cohen’s D. Negative effect sizes indicate lower HRQoL compared with normative population

^*^Indicates significant result

Normative population data as detailed in study by Varni et al. [48]

A summary of associations between participant clinical characteristics with both child and parent-reported PedsQL scores can be seen in Table 3. A significant difference was found in parent-reported PedsQL score across the different tumour groups (*p* = 0.041), with survivors of medulloblastoma recording significantly lower PedsQL scores than survivors in the other CNS tumours group (mean difference = -17.66, *p* = 0.036).

No significant associations were found between child-reported PedsQL score and the various participant characteristics.

### Cardiorespiratory fitness and health-related quality of life

A significant positive association was found between 6MWD and parent-reported PedsQL total score (r = 0.55, *p* < 0.001) and child-reported PedsQL total score (r = 0.48, *p* = 0.005). A similarly significant positive association was found between 6MWD and physical health (r = 0.50—0.63, *p* < 0.001 – *p* = 0.003) and also psychosocial health (r = 0.40—0.43, *p* = 0.012 – *p* = 0.023) as reported on the PedsQL.

## Discussion

We found that survivors of childhood CNS tumours in the first five years postcompletion of oncology treatment have lower levels of cardiorespiratory fitness and HRQoL compared to the general healthy paediatric population. Survivors of Medulloblastoma had lower cardiorespiratory fitness and HRQoL compared to survivors of other CNS tumours. We also found that higher levels of cardiorespiratory fitness were associated with higher levels of HRQoL. Understanding the severity of these impairments is important in order to design and develop targeted rehabilitation programmes to reduce the burden of these impairments.

Significantly lower levels of fitness have already been found in the wider population of survivors of childhood cancer when compared to non-cancer controls [16]. In our study, participants covered a mean 6MWD of 489.96 ± 61.48 m, which is significantly lower than the mean distance of 665 ± 84 m covered by Australian childhood cancer survivors in the study by Mizrahi et al. [19] (*p* < 0.001). The cohort of childhood cancer survivors in the Australian study however comprised predominantly of survivors of acute lymphoblastic leukaemia with no reference to how many survivors of CNS tumours were included, which may explain the variation in results [19]. Whilst the Australian childhood cancer survivors presented with low levels of cardiorespiratory fitness, the significant difference in mean 6MWD between our study population and the Australian population suggests that survivors of childhood CNS tumours present with lower levels of cardiorespiratory fitness than the wider population of childhood cancer survivors. Our findings are similar to those of Wolfe et al. [21], who reported impaired cardiorespiratory fitness amongst survivors of paediatric posterior fossa tumours. It is not fully understood why cardiorespiratory fitness is impaired amongst survivors of childhood CNS tumours. We know that survivors of childhood CNS tumours experience a high rate of neuromotor and physical function deficits including ataxia, impaired muscle power and reduced standing balance, which may influence their overall exercise capacity and performance during testing [17, 52].

Our study demonstrated that survivors of childhood CNS tumours experience significantly lower levels of HRQoL compared to healthy population norms. A systematic review by Schulte et al. [53] similarly found that survivors of childhood CNS tumours fared worse in terms of HRQoL when compared to healthy populations norms and also to survivors of non-CNS tumours. Our results further highlight that survivors of childhood CNS tumours experience long-term adverse psychosocial effects following the completion of their oncology treatment. There is a wide range of potential causes and explanations for why survivors of childhood CNS tumours experience lower HRQoL. This unique cohort of survivors are at increased risk for developing cognitive impairments and can experience difficulties with hearing, vision, growth and physical functioning, all of which can negatively influence overall HRQoL [52, 54].

Our study found higher levels of cardiorespiratory fitness were associated with higher levels of HRQoL in survivors of childhood CNS tumours. This contrasts with the findings of Kuhn et al. [55], who found no association between cardiorespiratory fitness and HRQoL. However the study by Kuhn et al. [55] included survivors of a variety of different childhood cancers and reported that twelve participants were diagnosed with solid tumours, with no further breakdown within this of how many of these solid tumours were CNS tumours. Therefore the positive association between cardiorespiratory fitness and HRQoL may be uniquely present in survivors of childhood CNS tumours. However it is not possible from our study to suggest any explanations for why this association may be uniquely present in survivors of childhood CNS tumours. The work by Cox et al. [56] and Riggs et al. [57] may however provide a potential explanation. These authors reported results from the same trial which found that exercise improves cognition and promotes white matter and hippocampal growth thereby enhancing neural recovery in survivors of childhood CNS tumours. These exercise-induced improvements have the potential to positively influence HRQoL.

Whilst our results suggest that assessment and monitoring of cardiorespiratory fitness and HRQoL in all survivors of childhood CNS tumours would be beneficial, survivors of Medulloblastoma were found to have significantly lower levels of cardiorespiratory fitness and HRQoL than survivors of other CNS tumours and therefore should be prioritised for regular monitoring. Future research needs to focus on intervention strategies to improve both the physical and psychosocial late-effects of childhood CNS tumour treatment. Our study found that higher levels of cardiorespiratory fitness were associated with higher levels of HRQoL, which raises the question of whether exercise and physical activity interventions are the key to addressing both the physical and psychosocial late-effects experienced by survivors of childhood CNS tumours?

A recent systematic review found that therapeutic exercise is a potentially safe and feasible intervention for survivors of childhood CNS tumour to improve their QoL and wellbeing [58]. Large-scale intervention studies are still required to examine the efficacy of exercise for improving overall health outcomes in survivors of childhood CNS tumours. This research would help determine the best timing, setting and type of exercise interventions for survivors of childhood CNS tumours. Particular emphasis should be placed on assessing the feasibility and benefits of exercise interventions at different timepoints throughout the cancer journey to determine whether supervised exercise interventions led by exercise professionals during treatment could reduce the severity of cancer-related late-effects and even whether it could play a protective or preventative role against these.

This is the only national dataset of cardiorespiratory fitness and HRQoL outcomes in Irish survivors of childhood CNS tumours. We had a high recruitment rate (only 11% of eligible participants declined), which minimises selection bias. Our use of objective measures for the measurement of cardiorespiratory fitness and HRQoL is a particular strength of this study, allowing our results to be more accurately compared to other similar studies within this population and also within the wider population of childhood cancer survivors.

There were several limitations in the current study. These include the cross-sectional design which limits the ability to draw causal inferences and the relatively small sample size which means results should be interpreted with caution.

## Implications for cancer survivors

In conclusion, we have identified that survivors of childhood CNS tumours in their first five years post treatment present with impaired cardiorespiratory fitness and HRQoL when compared to healthy samples from other published studies. Our results highlight the need to research intervention strategies that can be adopted effectively into clinical practice to improve the quality of survivorship for survivors of childhood CNS tumours.

## Supplementary Information

Below is the link to the electronic supplementary material.

Supplementary file1 (PDF 214 KB)

## Data Availability

The data that support the findings of this study are not openly available due to reasons of sensitivity and are available from the corresponding author upon reasonable request.
