# Supplementary material for: Cardiorespiratory fitness and health-related quality of life in survivors of childhood central nervous system tumours
Source: Support Care Cancer. 2023 Jun 15;31(7):395. doi: 10.1007/s00520-023-07854-9 (PMC10272264; doi:10.1007/s00520-023-07854-9)
Supplement: Supplementary file 1 — Supplementary file1 (PDF 214 KB) [file 520_2023_7854_MOESM1_ESM.pdf]

Supplementary Information

Article title: Cardiorespiratory fitness and health-related quality of life in survivors of childhood central nervous system tumours.

Journal name: Journal of Supportive Care in Cancer

Author names: Ms. Rachael Keating[1,2], Dr. Sarah Curry and Prof. Juliette Hussey.

Affiliation and email address of corresponding author:

[1] School of Medicine, Trinity College Dublin, University of Dublin, Dublin, Ireland.

[2] Physiotherapy Department, Children's Health Ireland at Crumlin, Dublin, Ireland

Email: keatinra@tcd.ie

ONLINE RESOURCE TABLE 1 Equations to predict the 6 minute walk distance in children and adolescents

**Model Population**	**Age-adjusted**<br><br>**(age in years (y))**
**Males**	
**<13 y**	24.18*y + 385.18
**≥13 y**	13.08*y + 476.69
**Females**	
**<12 y**	20.83*y + 413.94
**≥12 y**	-8.66*y + 757.42
**Abbreviations:** *multiplied by, *y* years.<br><br>Equations extracted from Ulrich, S., et al., *Reference values for the 6-minute walk test in healthy children and adolescents in Switzerland.* BMC Pulm Med, 2013. **13**: p. 49.	
